# P-1390. Accuracy of computer-aided detection of chest x-rays for pulmonary tuberculosis among adults with Xpert Ultra trace-positive sputum

**DOI:** 10.1093/ofid/ofaf695.1577

**Published:** 2026-01-11

**Authors:** Joowhan Sung, Annet Nalutaaya, Ronit Dalmat, Caitlin Visek, Mariam Nantale, James Mukiibi, Patrick Biché, Gabrielle Stein, Achilles Katamba, Douglas Wilson, Paul K Drain, Emily A Kendall

**Affiliations:** Johns Hopkins University School of Medicine, Baltimore, MD; Walimu, Kampala, Kampala, Uganda; University of Washington, Seattle, Washington; Johns Hopkins University, Baltimore, Maryland; Walimu, Kampala, Kampala, Uganda; Walimu, Kampala, Kampala, Uganda; Johns Hopkins Bloomberg School of Public Health, Baltimore, Maryland; University of Washington, Seattle, Washington; Makerere University College of Health Sciences, Kampala, Kampala, Uganda; Harry Gwala Regional Hospital, University of KwaZulu-Natal, Pietermaritzburg, KwaZulu-Natal, South Africa; University of Washington, Seattle, Washington; Johns Hopkins University School of Medicine, Baltimore, MD

## Abstract

**Background:**

The clinical significance of low-level positive results from molecular testing of sputum for tuberculosis (TB) remains uncertain, and additional diagnostic testing, such as chest X-ray, might help to guide patient care. In high-TB-burden settings, where radiologists are often unavailable, artificial intelligence-based computer-aided detection (CAD) may be used to analyze chest X-rays, but its accuracy in this context remains unclear.

**Table 1.**
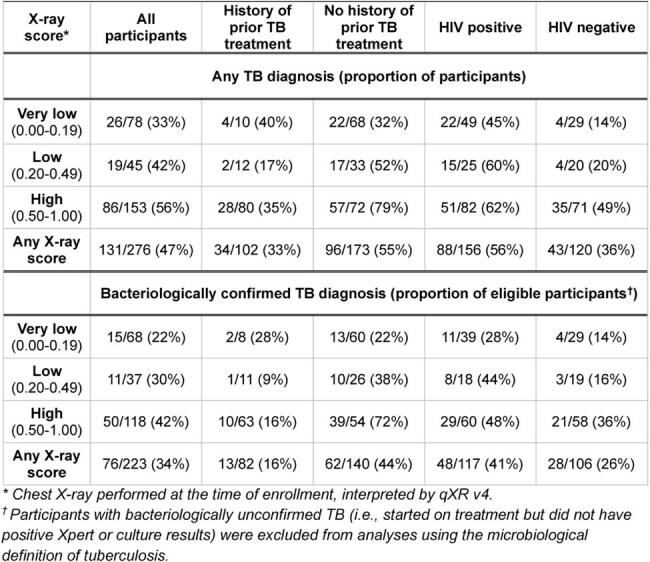
TB status determination at three months among outpatients with Xpert Ultra trace-positive sputum – overall and by X-ray score category and/or clinical risk subgroup, using two definitions of TB based on treatment decision with or without bacteriological confirmation.

**Figure 1.**
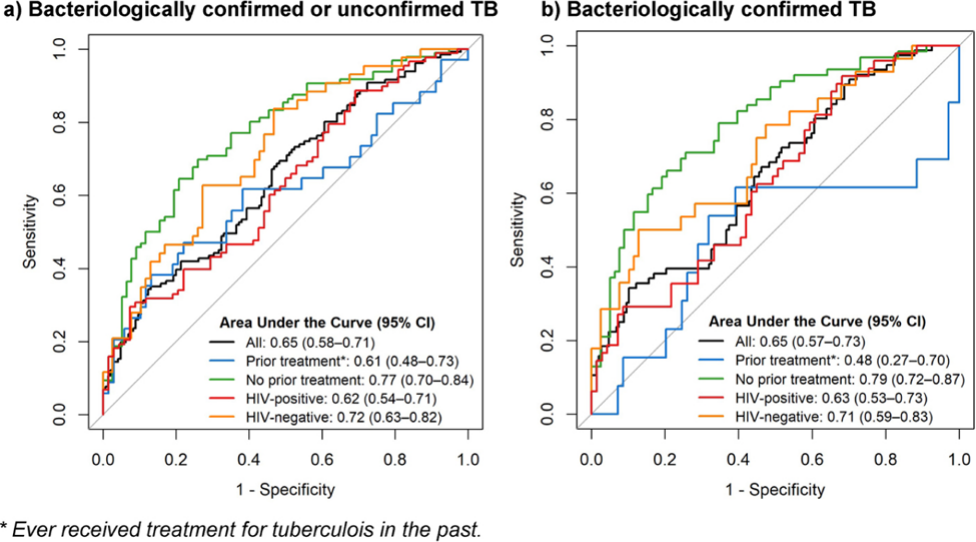
Receiver operating characteristic curves showing the performance of computer-aided detection software (qXR v4) on a baseline X-ray, for classifying bacteriologically confirmed and/or unconfirmed tuberculosis among individuals with an initial trace-positive sputum Xpert Ultra diagnostic result and up to three months of further diagnostic evaluation.

**Methods:**

We evaluated CAD accuracy among adults who presented to clinics in Uganda and South Africa and tested trace-positive on sputum Xpert Ultra (“Xpert”). Participants underwent repeat sputum Xpert, two mycobacterial cultures, chest X-ray, and HIV testing, and those not started on treatment after this baseline evaluation repeated sputum Xpert and culture testing at 1 and 3 months to further clarify TB status. Chest X-ray images were retrospectively analyzed by CAD software (qXR v4, Qure.ai). We evaluated CAD accuracy compared to two TB definitions: a composite definition that included clinical diagnoses, and a second requiring bacteriological confirmation.

**Figure 2.**
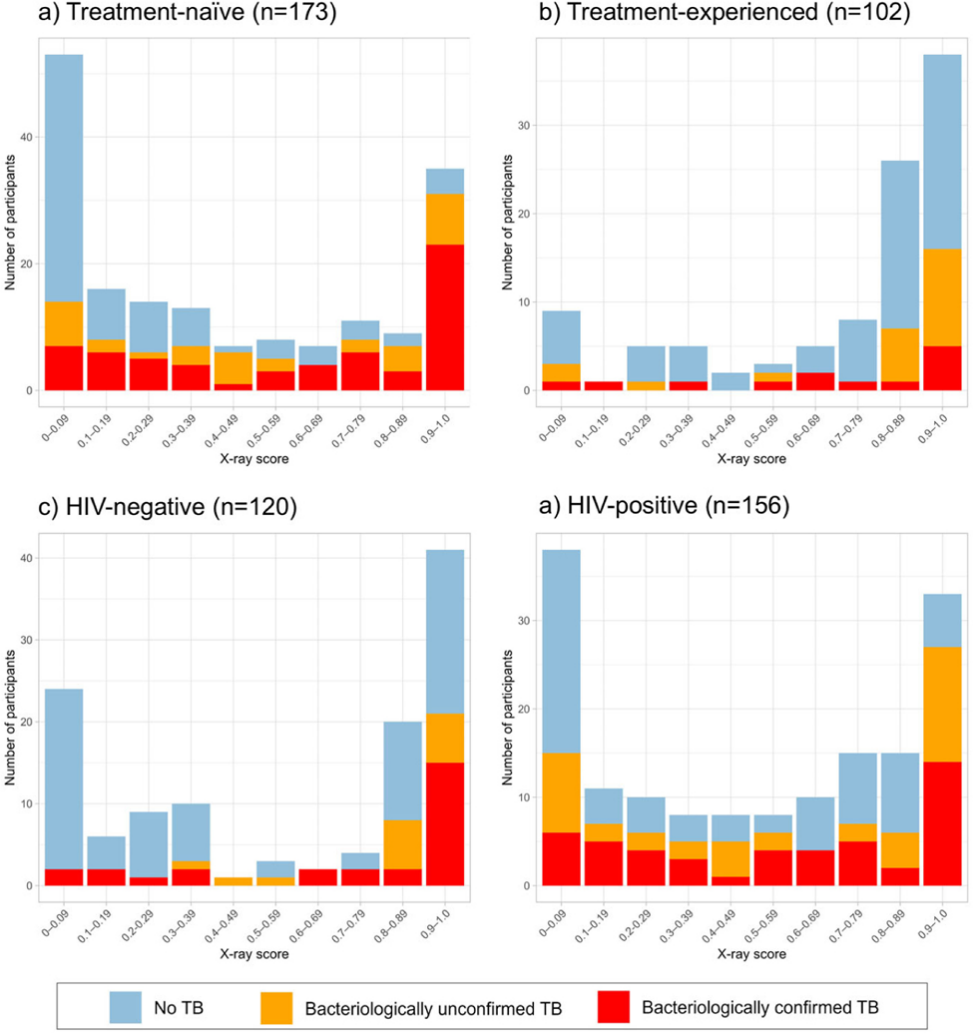
Distribution of X-ray scores from computer-aided detection software (qXR v4), among individuals with trace-positive sputum at clinics in Uganda and South Africa, colored by TB diagnostic outcomes and stratified by history of previous tuberculosis treatment and by HIV status.

**Results:**

Among 276 participants, median age was 37 years (interquartile range 30-46), 141 (51%) were female, 156 (57%) were HIV-positive, and 102 (37%) had prior treatment for TB. The median X-ray score was 0.63 (interquartile range 0.14-0.91), and 153 (55%) had a score above 0.5 (the manufacturer-recommended threshold). TB was diagnosed in 131 patients (47%), including 76 with bacteriological confirmation. CAD had an area under the curve (AUC) of 0.65 (95% confidence interval 0.58–0.71) using a composite definition and 0.65 (0.57–0.73) for bacteriologically confirmed TB. An X-ray score threshold of 0.5 had sensitivity and specificity of 66% and 54%, respectively, for bacteriologically confirmed TB. Lowering the threshold to 0.2 increased sensitivity to 80% but decreased specificity to 37%. The AUC of CAD was higher among patients without prior TB treatment and among those without HIV infection (Figure 1).

**Conclusion:**

Chest x-ray with CAD could serve as a supplementary tool for diagnosing TB in adults with Xpert trace-positive sputum, particularly those without prior tuberculosis or HIV infection. However, a low X-ray score did not reliably rule out TB disease.

**Disclosures:**

Paul K. Drain, MD, MPH, Abbott Diagnostics: Advisor/Consultant|Abbott Diagnostics: Grant/Research Support|Giner: Advisor/Consultant|OraSure: Advisor/Consultant|Revvity: Advisor/Consultant|Revvity: Grant/Research Support|Roche: Advisor/Consultant|Roche: Grant/Research Support

